# Patients’ perceptions on temporomandibular disorder treatment with hydrostatic oral splints - a pilot study

**DOI:** 10.1038/s41405-022-00096-7

**Published:** 2022-02-05

**Authors:** H. Sabbagh, A. Sabbagh, A. Heppner, C. Auer, A. Wichelhaus, L. Hoffmann

**Affiliations:** 1 Senior Physician, Department of Orthodontics and Dentofacial Orthopedics, University Hospital, LMU Munich, Munich, Germany; 2 DMD, Private Orthodontic Practice, Erlangen, Germany; 3 DMD, Private Orthodontic Practice, Potsdam, Germany; 4 Professor and Chair, DMD, Department of Orthodontics and Dentofacial Orthopedics, University Hospital, LMU Munich, Munich, Germany; 5 Assistant Physician, Department of Orthodontics and Dentofacial Orthopedics, University Hospital, LMU Munich, Munich, Germany

**Keywords:** Temporomandibular disorders, Orofacial pain

## Abstract

**Objective:**

To evaluate temporomandibular disorder (TMD) treatment with a prefabricated, hydrostatic oral splint (HOS) based on self-reported patient’s symptoms using a standardized questionnaire.

**Methods:**

Two hundred fifty-eight questionnaires from patients diagnosed with TMD and subsequently treated with HOS were collected from two independent private practices. Based on patient’s comfort the questionnaire recorded TMD symptoms and symptom regression. Descriptive and comparative statistics was carried out using SPSS.

**Results:**

A total of 221 questionnaires were analyzed. Patients reported TMD symptoms such as pain (93.2%), TMJ clicking (66.1%), headache (25.8%), cervical spine disorders (23.5%), restricted mouth opening (22.6%) and tinnitus (11.8%). For most symptoms, improvement was reported mostly after two weeks, except for tinnitus, where positive effects were usually reported after four weeks.

**Conclusion:**

HOS seem to be effective for immediate treatment of pain and other TMD symptoms. Based on the available data, a treatment period of four weeks can be recommended.

## Introduction

Temporomandibular disorders (TMDs) imply a wide range of clinical patterns, including temporomandibular joint dysfunction, masticatory muscle disorders and disorders of associated structures [1, 2]. The most common symptoms range from joint and muscle pain, headache, joint noise, limited mouth opening, facial pain to cervical-spine disorders [3, 4]. Even otological symptoms like tinnitus, earache, dizziness and hypo- or hyperacusis can be related to TMDs [5]. With a prevalence between 10% and 15%, TMDs represent the most common cause of non-tooth related pain in the oro-facial region [6].

TMDs belong to the group of orofacial-pain conditions and exhibits a multifactorial etiology [7, 8]. Contrary to initial presumptions, occlusal factors play a minor role in the development of TMDs [9]. Nevertheless, a significant number of patients relate TMD symptoms with occlusal factors and therefore consult a dentist or orthodontist at first when symptoms occur [10]. According to the current state of art, biological, psychological and social factors are crucial for the pathogenesis of TMD [11].

Due to the variety of symptoms and the multifactorial etiology, a standardized treatment concept is not available [8]. A variety of different treatment concepts have been described, which can be divided into invasive and non-invasive approaches. Invasive treatment concepts include irreversible occlusal changes and surgical interventions. Several publications have shown that non-invasive, reversible approaches have advantages over invasive therapies in terms of efficacy and risks [12, 13]. Non-invasive therapeutic approaches include observation, counseling, occlusal splint therapy, physical therapy, pharmacotherapy, behavioral therapy or a combination of several approaches, so-called multimodal treatment [12, 13].

Occlusal splint therapy represents the most common treatment modality for TMDs [8]. There are several studies that postulate the efficacy of these occlusal splints to reduce pain and other TMD symptoms [13–17]. In particular, patients with myogenous TMDs can have a significant benefit from occlusal splint therapy [18]. However to date, the mechanisms of action are still not completely understood [11].

Various types of occlusal splints are used for the treatment of TMD symptoms. While the terminology is not consistent, occlusal splints can be divided into the following groups: non-occluding splints, stabilization splints, prefabricated splints, anterior bite splints and anterior repositioning splints [16]. Stabilization splints are the type most frequently used [19].

Compared to stabilization splints, Hydrostatic oral splints (HOS) represent a relatively new occlusal splint system. HOS are prefabricated, splints that contain two water filled pads, connected with a tube for hydrostatic balance of both temporomandibular joints and disclusion of the dental arches. The main advantage of HOS is the possibility of immediate treatment, even in cases with limited mouth opening [20]. Furthermore, jaw and joint position are determined and balanced by the water filled pads. Thus, occlusal adjustment of the appliance is not required. However, to date, there are only few studies on the treatment with HOS and the effectiveness is currently unknown [20].

Therefore, the aim of this study was to evaluate an established treatment protocol for TMD treatment with a prefabricated, hydrostatic oral splint based on the patient’s comfort using standardized questionnaires.

## Material and methods

### Overview

This retrospective study analyzed questionnaires collected from two independent orthodontic practices which were completed by consecutive patients between 2011 and 2020. Due to the retrospective design, written informed consent was not obtained. It was ensured that the evaluation of all data was anonymized by entering all data in a separate Excel file and by giving each patient a randomly assigned number. Thus, reidentification of the patient’s name was not possible. The study protocol was approved by the Ethics Committee of the LMU University Hospital (ref. no.: 20–467 KB).

### Recruitment, clinical examination and treatment

**Fig. 1:**
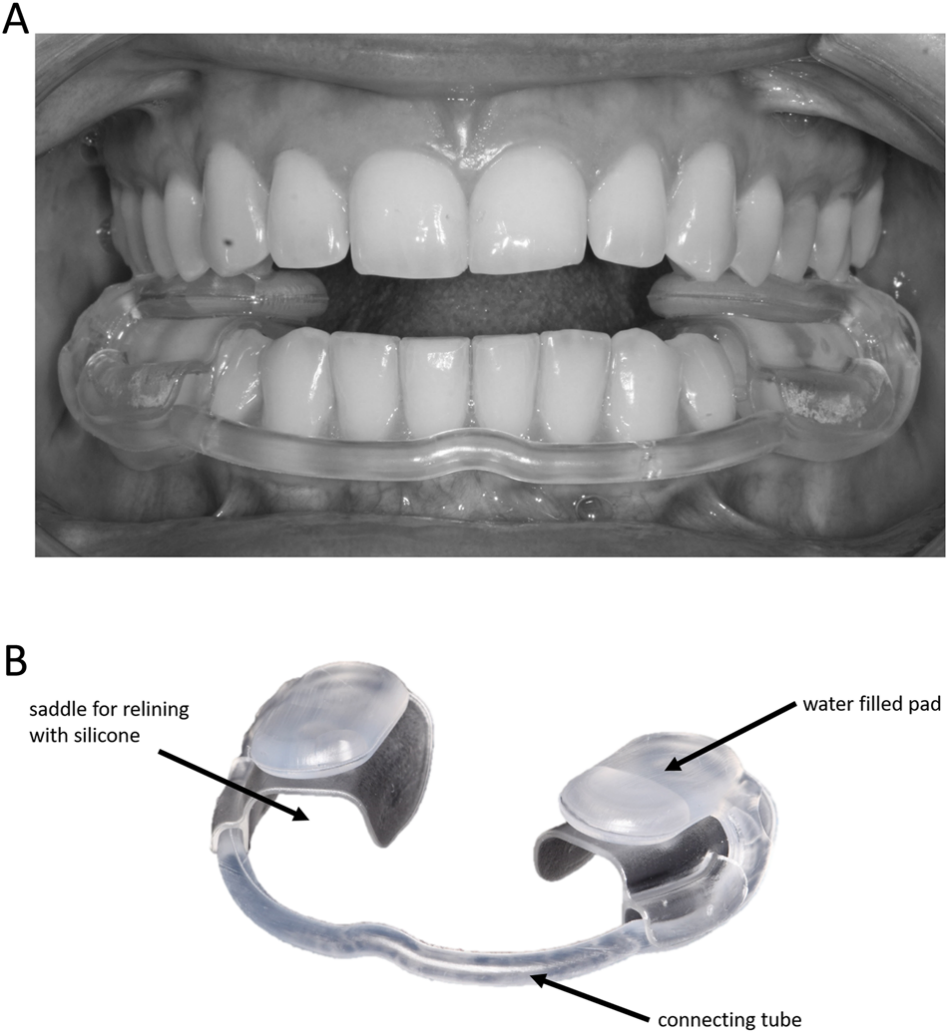
Hydrostatic oral splint. **A** Clinically adjusted appliance. The dental arches are discluded by the water filled pads. **B** Design and components of the appliance.

All patients who received a treatment due to TMD symptoms were examined by an experienced clinician (A.S and A.H) with >20 years’ experience in TMD treatment. Medical history was obtained for each patient, followed by a clinical examination and functional analysis according to the guidelines of the German Society for Functional Diagnostics and Therapy [21]. This analysis includes a detailed pain-related history, palpation and auscultation of the temporomandibular joint, palpation of the masticatory muscles, examination of lower jaw movements, upper/lower jaw relationship, occlusion, joint function and mouth opening. Patients diagnosed with TMD received treatment with a hydrostatic oral splint (Aquasplint, Teledenta GmbH, Chemnitz, Germany; Fig. 1a, b).

Patients of both genders with with acute, pain-related TMD or headache (Myalgia, Myofascial pain, Arthralgia, Headache attributed to TMD) and sufficient dental support for HOS (presence of at least two premolars per side) were included.

Restricted mouth opening (<40 mm or less than the width of three fingers), limitation of jaw movement or pain during jaw movement or chewing were considered as dysfunction. Patients with chronic pain, history of trauma or congenital syndromes were not included in the study. Pain was regarded chronic if it was reported for >3 months [22].

Patients were asked to wear the splint at least for ten hours per day (two hours a day and eight hours a night) for a period of at least four weeks, as recommended by the manufacturer [23].

### Questionnaire

A standardized questionnaire, developed by Gedrange et al. was handed out to the patient together with the HOS [24]. The patient were asked to complete the questionnaire until the next follow-up appointment (after 6 weeks). The questionnaire was designed to provide systematic feedback to the practitioner on the patient’s perception of treatment. The questionnaire included the wearing period (daily, nightly, total), symptoms (pain, temporomandibular clicking, restricted moth opening, cervical spine syndrome, tinnitus), symptom improvement in percent (0%, 30%, 60%, 90%, 100%) and time required for improvement (1 day, 1 weeks, 2 weeks, 3 weeks or later) (Appendix. 1). The questionnaire was designed that only predefined answers could be marked. In 2014, the questionnaire was extended by 4 questions to gain more detailed insight into TMD related symptoms.

### Statistics

Only fully filled questionnaires from patients who completed treatment with HOS were evaluated. Statistical analysis was performed using SPSS statistics version 26.0 for Windows (SPSS Inc., Chicago, USA). Descriptive statistic was carried out to describe the frequencies and Pearson’s Chi Square test was used to determine significant differences. A *p* value of 0.05 was considered significant.

## Results

**Fig. 2:**
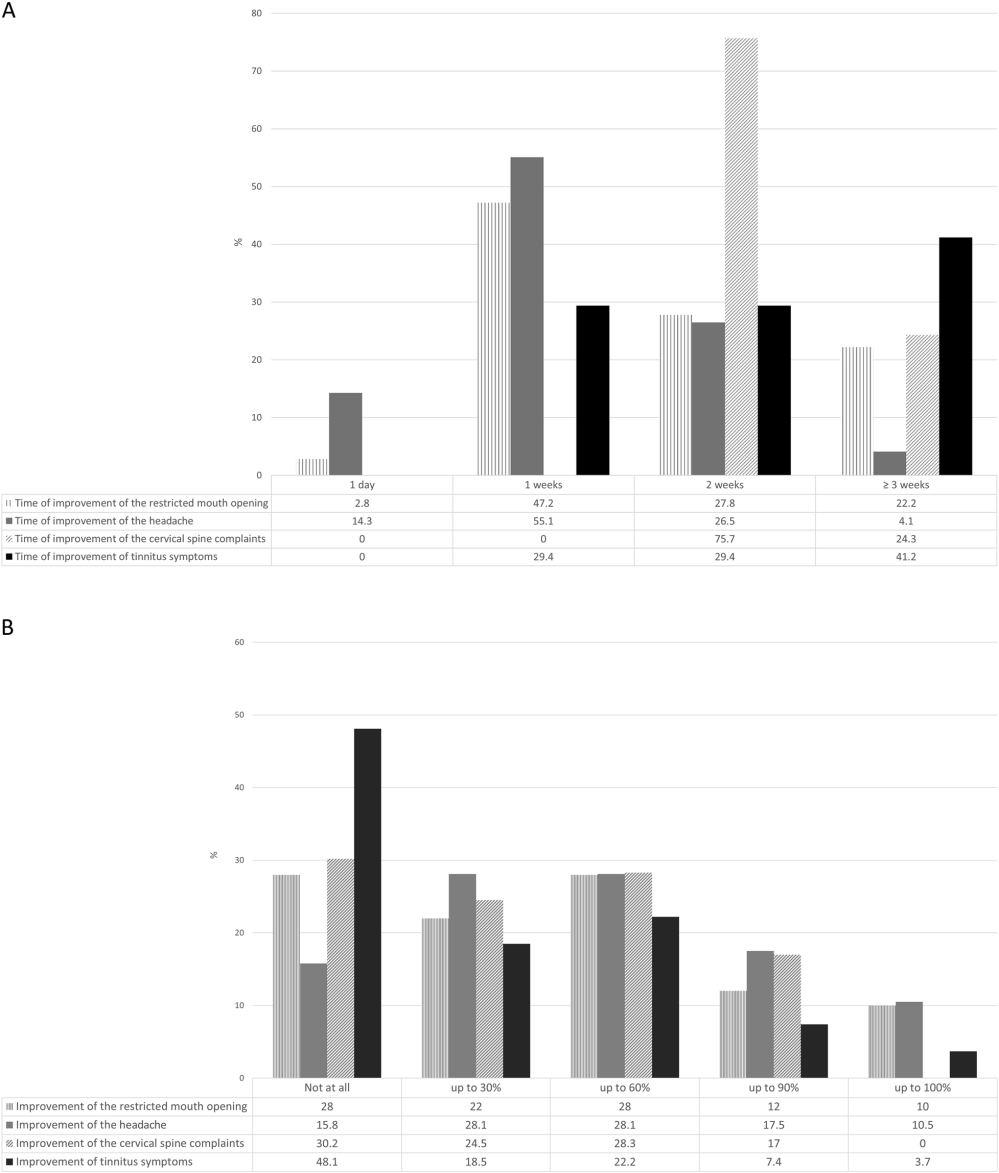
Results of the analyzed questionnaire. **A** Frequency of subjective symptom improvements with regard to the time course of treatment. **B** Extent of subjective symptom improvement for the different symptoms.

Two hundred-fifty-eight questionnaires were analyzed in the study. Thirty-seven questionnaires were excluded, primarily because of an incomplete, illegible, or unclear filling out of the questionnaire. A total of 221 questionnaires remained for evaluation, 73 (30.0%) questionnaires of the primary version and 148 (67.9%) of the extended version. The study population consisted of 158 (71.5%) women and 39 (17.6%) men. Twenty-four patients (10.9%) did not report gender. Results for the different items of the questionaire are shown in Table 1 and Fig. 2.

**Table 1:** Results of the analyzed questionnaire.

	Answer	%
Total wearing period	<3 weeks	17.2
	3 weeks	11.3
	4 weeks	24.9
	5 weeks	11.8
	>5 weeks	34.8
Daily wearing period	0 h	41.2
	1 h	30.8
	2 h	22.2
	3 h	3.2
	>3 h	2.7
Nightly wearing period	≤6 h	15.4
	7 h	26.2
	8 h	43
	9 h	10
	10 h	5.4
Pain	Yes	93.2
	No	6.8
Pain reduction	Not at all	9.7
	30%	25.2
	60%	33.5
	90%	24.3
	100%	7.3
Time of pain reduction	1 day	13.1
	1 weeks	48.5
	2 weeks	22.3
	≥3 weeks	16
Temporomandibular joint clicking	Yes	66.1
	No	33.9
Reduction of temporomandibular joint clicking	Not at all	31
	30%	21.4
	60%	27.6
	90%	17.9
	100%	2.1
Time of reduction of temporomandibular joint clicking	1 day	7.9
	1 weeks	41.6
	2 weeks	30.7
	≥3 weeks	19.8
Types of symptoms	Restricted mouth opening	22.6
	Headaches	25.8
	Cervical spine complaints	23.5
	Tinnitus	11.8
	No symptoms	16.3

### Symptom prevalence

Regarding TMD symptoms, patients most frequently reported pain (93.2%) followed by temporomandibular joint clicking (TMJ) clicking (66.1%), headaches (25.8%), cervical spine disorders (23.5%), restricted mouth opening (22.6%) and tinnitus (11.8%). Seventeen percent of the patients described none of these symptoms (Tab. 1).

### Total and daily wearing period

Most of the patients (71.5%) wore the HOS for the recommended time period of 4 or more weeks (Tab. 1). During the day, the splint was mostly not used (41.2%, 0 h) or used for one or two hours (53.0%). As recommended patients wore the splint for 8 or more hours during the night (58.4%).

### Symptom changes

Ninety percent of the patients reported an improvement of pain symptoms. Most patients reported an improvement of pain starting after 1 week (48.5%) most frequently rated as a 60% reduction (33.5%) (Fig. 2a, b), whereas 9.7% of the patients did not experience any pain improvement. About 72% of the patients reported on an improvement of a restricted mouth opening mostly rated as a 60% reduction of symptoms. Eighty-four percent of patients with headache exhibited an improvement of symptoms, mainly rated as a 30% or 60% reduction (each 28.1%). A 90% reduction of headache symptoms was reported by 17.5% of the patients. Regarding cervical spine disorders, 69.8% noticed a reduction of their symptoms. Half of the patients (51.9%) experienced an improvement of tinnitus symptoms, mostly starting after 4 weeks (41.2%). In addition, 69.0% of the patients reported on a reduction of TMJ clicking, most frequently rated as a 60% reduction of symptoms (27.6%) (Fig. 2a, b).

In the majority of patients the improvement of all evaluated symptoms started after one week or two weeks after starting HOS treatment (Fig. 2a, b). By contrast, in patients with tinnitus symptoms improvement was reported only after 4 weeks in 41.2% of the patients.

Statistical analysis showed no significant association between wearing duration and pain or symptom reduction, and no significant association between gender and pain and symptom reduction (*p* > 0.05).

## Discussion

### Discussion of results

There are only few studies on the treatment and efficacy of HOS. Mostly case reports and descriptions of the appliance are published [25–27]. Giannakopoulos et al. investigated the effectiveness of HOS in a randomized, controlled pilot trial. However, only 12 patients per treatment group were included. The authors stated that HOS seemed to be effective for reducing the pain intensity. However, sample size was too small to draw meaningful conclusions [20].

In this study a total of 221 questionnaires from patients undergoing TMD treatment with HOS were evaluated. With a gender distribution of 158 women (71.5%) and 39 men (17.6%) this study included a higher proportion of women (ratio of 4.1:1 women:men) compared to previous epidemiological studies (3:1 women:men) [28, 29]. Bueno et al. analyzed gender differences in temporomandibular disorders in a systematic review and meta analysis [29]. They found that women are twice as likely to develop TMDs compared to men. To date, it is not known what predisposes women to develop TMDs more often than men. Most likely, hormonal differences, cultural and social factors, higher levels of occupational stress, and differences in pain perception may be possible causes [30–32]. Although the study included more women than men with a TMD symptomatology, no significant differences between men and women regarding the frequency of TMD symptoms or symptom reduction were observed.

Most patients (71.5%) wore the HOS as recommended (minimum 4 weeks). The treatment period with HOS alone should not exceed six weeks because undesirable occlusal effects such as over-eruption of the unopposed teeth may occur, as the HOS covers only parts of the dentition [9]. If HOS are used for medium-term treatment (>6 weeks), vacuum-formed retainers should be used additionally to avoid over-eruption and adverse effects. Nevertheless, longer wearing periods over 6 weeks are questionable. Results of the study documented an improvement of most symptoms already after one week or two weeks. Only tinnitus showed an improvement only after 4 weeks in most of the cases. Thus, the use of HOS seems at least to be effective for immediate and short-term treatment (4–6 weeks), especially for the most common signs and symptoms of TMDs. After the initial treatment, reexamination and evaluation of the treatment are necessary to decide whether the therapy should be terminated or continued. For medium- or long-term treatment other therapeutic options may be considered depending on the medical findings.

Pain is the most frequently reported symptom of patients suffering from TMDs [33–35]. These findings are in line with the study, observing self-reported pain in 93.2% of the patients. Ninety percent of the patients treated with HOS experienced an improvement of pain symptoms most frequently rated as a 60% reduction (33.5%). Improvement of pain following TMD treatment may be explained by reduced muscle activity and a redirection of forces within the joint [11]. Unlike with stabilizing splints made of hard acrylic plastic, HOS create a counterforce when the water-filled pads are compressed by masticatory activity. These counterforces may inhibit bruxism patterns and prevent the development of excessive masticatory forces that could lead to pain in the joint and muscles.

In this study TMJ clicking was the second most frequently reported symptom (66.1%), a finding in line with previous studies [35]. Joint clicking can be disturbing for patients and cause discomfort, but often presents without any other signs of dysfunction or pain [34]. TMJ clicking is considered to be mainly related to anterior disk displacement with reduction [36, 37]. In this study, 69.0% of the patients reported on an improvement in TMJ clicking, mostly starting after 1 week of treatment. The water-filled pads of the HOS prevent tooth-to-tooth contact and thereby change the position of the mandible. It is likely that change of the positions of the temporomandibular joints and discs with HOS treatment explains the beneficial effect with regard to TMJ clicking. Similar effects have already been observed using other types of occlusal splints [38]. The authors suggested that improvement of clicking noise may be attributable to morphological changes of the disc itself, eliminating the physical barrier for jaw translation and thus decreasing the sound intensity of TMJ clicking.

Previous studies have shown that headache and TMDs are comorbidities [39, 40]. In this study, headache was reported less frequently (25.4%) than in other studies that examined patients with TMD symptoms, showing a prevalence of headache ranging from 79.3% to 87.0% [34, 35]. It is presumed that the association of headache and TMDs is related to common nociceptive pathways [40]. Thus, improvement of TMD related pain may concomitantly lead to a reduction of headaches. This mechanism may also explain a self-reported 30% reduction of headache symptoms by 84.2% of the patients.

In previous studies the prevalence of otological symptoms of patients with TMDs ranges between 35.8% to 82.4% [35, 41]. In this study the observed frequency of tinnitus was substantially lower (11.8%). Association between TMDs and tinnitus is explained by the close anatomical and functional connections of the somatosensory system of the head and the auditory system in the extended spinal chord [42]. It has been shown that TMD treatment can contribute to a reduction or complete remission of otological symptoms, although there is no sufficient evidence for a cause-effect relationship [5]. Several studies reported positive effects on otological symptoms including tinnitus after conservative therapy of TMDs with occlusal splints [43–45] without being able to provide conclusive or differentiated explanations. In this present study, symptom improvement was reported by 51.9% of the patients with tinnitus. In contrast to the other symptoms investigated improvement most frequently was reported after 4 weeks of HOS treatment. Most likely not the therapy with occlusal splints contributed to the reduction of tinnitus, but the reduction of other TMD symptoms. In turn, the decrease of the other symptoms occurred only after 2 weeks. Therefore, a tretment period of four weeks can be suggested for patients with tinnitus. However, there are various other causes for tinnitus that are not related to TMDs. Thus, interdisciplinary investigations should always be performed to clarify the cause.

Patients with restricted mouth opening (72.0%) reported an improvement of their complaints mostly rated as a 60% reduction. Pain reduction, relaxation of the associated muscles, influence on neuromuscular activity and subsequent joint relief could have led to the improvement [46, 47]. In contrast to other subjective symptoms like pain and headache, the assessment of the mouth opening can be objectified by determining the distance between incisal edges. Although these data was collected clinically, this study only evaluated the questionnaires and not the patients clinical data.

There is no universally applicable treatment concept for TMDs [47]. The spectrum of symptoms, etiological and individual factors make it difficult to standardize treatment to a high degree. Regarding short-term treatment effects, occlusal splints seem to be equally effective in reducing pain as other treatment options like physical therapy, acupuncture and behavioral therapy [48]. Multimodal treatment concepts seem to be more beneficial than single treatment approaches [8]. The investigation of the most appropriate treatment modalities for different TMD sub-groups represents a main target for future researches [8]. However, non-invasive, reversible and cost-effective treatment concepts should be prefered [49–51].

Further, prospective studies are necessary to investigate the treatment effects of HOS, especially in comparison with other occlusal appliances and in combination with other treatment modalities. The main benefit of HOS is the possibility of immediate treatment, also representing a more cost-effective and time-effective method compared to stabilization splint therapy.

### Limitations

This is a retrospective evaluation of self-reported patients complaints associated with TMDs, documented on the basis of a questionnaire, allowing assignment to rather broad categories of response options. There was no control group, as the study included patients of two private practices following a consistent TMD therapy concept using HOS. However, results of this retrospective analysis may give valuable advice to practitioners about patients’ perceptions following treatment of TMDs with HOS and appropriate treatment period.

Clinical functional analysis as it was performed in this study (according to the guidelines of the German Society for Functional Diagnostics and Therapy) is a standard method for TMD diagnosis [8]. The Diagnostic Criteria for Temporomandibular Disorder (DC/TMD) represents a more detailed, evidence-based tool for TMD diagnosis. However the examination procedure is complex and clinicians have to be trained and calibrated. Thus, this method is rarely used in private clinics and practices, but rather in university hospitals and specialized centers.

## Conclusions

The findings suggest that HOS can be effective for treatment of pain and other TMD symptoms. It appears, that most beneficial treatment effects occurred after two weeks at the latest after starting HOS treatment, with the exception of patients with tinnitus, in whom improvement was most commonly reported after 4 weeks. Further studies are necessary to prospectively investigate the efficacy of hydrostatic oral splints in comparison with other therapeutic measures.

## Supplementary information

Appendix 1

## References

[1] Peck CC, Goulet JP, Lobbezoo F, Schiffman EL, Alstergren P, Anderson GC (2014). Expanding the taxonomy of the diagnostic criteria for temporomandibular disorders. J Oral Rehabil.

[2] Schiffman E, Ohrbach R, Truelove E, Anderson G, Goulet JP, List T (2014). Diagnostic criteria for temporomandibular disorders (DC/TMD) for clinical and research applications: recommendations of the international RDC/TMD Consortium Network* and Orofacial Pain Special Interest Group†. J Oral Facial Pain Headache.

[3] Al-Hasson HK, Ismail AI, Ash MM (1986). Concerns of patients seeking treatment for TMJ dysfunction. J Prosthet Dent.

[4] Wiesinger B, Malker H, Englund E, Wanman A (2009). Does a dose-response relation exist between spinal pain and temporomandibular disorders?. BMC Musculoskelet Disord.

[5] Kusdra PM, Stechman-Neto J, Leão BLC, Martins PFA, Lacerda ABM, Zeigelboim BS (2018). Relationship between otological symptoms and TMD. Int Tinnitus J.

[6] Macfarlane TV, Glenny AM, Worthington HV (2001). Systematic review of population-based epidemiological studies of oro-facial pain. J Dent.

[7] Dworkin SF (1994). Perspectives on the interaction of biological, psychological and social factors in TMD. J Am Dent Assoc.

[8] Manfredini D, Bucci MB, Montagna F, Guarda-Nardini L (2011). Temporomandibular disorders assessment: medicolegal considerations in the evidence-based era. J Oral Rehabil.

[9] Manfredini D, Lombardo L, Siciliani G (2017). Temporomandibular disorders and dental occlusion. A systematic review of association studies: end of an era?. J Oral Rehabil.

[10] Chung J, Lobbezoo F, van Selms MKA, Chattrattrai T, Aarab G, Mitrirattanakul S (2020). Physical, psychological and socio-demographic predictors related to patients’ self-belief of their temporomandibular disorders’ aetiology. J Oral Rehabil.

[11] Klasser GD, Greene CS (2009). Oral appliances in the management of temporomandibular disorders. Oral Surg Oral Med Oral Pathol Oral Radio Endod.

[12] Michelotti A, Iodice G (2010). The role of orthodontics in temporomandibular disorders. J Oral Rehabil.

[13] List T, Axelsson S (2010). Management of TMD: evidence from systematic reviews and meta-analyses. J Oral Rehabil.

[14] Fricton J, Look JO, Wright E, Alencar FG, Chen H, Lang M (2010). Systematic review and meta-analysis of randomized controlled trials evaluating intraoral orthopedic appliances for temporomandibular disorders. J Orofac Pain.

[15] Ebrahim S, Montoya L, Busse JW, Carrasco-Labra A, Guyatt GH (2012). The effectiveness of splint therapy in patients with temporomandibular disorders: a systematic review and meta-analysis. J Am Dent Assoc.

[16] Al-Moraissi EA, Farea R, Qasem KA, Al-Wedeai MS, Al-Sabahi ME, Al-Iryani GM. Effectiveness of occlusal splint therapy in the management of temporomandibular disorders: network meta-analysis of randomized controlled trials. Int J Oral Maxillofac Surg. 2020. 10.1016/j.ijom.2020.01.004. 10.1016/j.ijom.2020.01.004 31982236

[17] Forssell H, Kalso E (2004). Application of principles of evidence-based medicine to occlusal treatment for temporomandibular disorders: are there lessons to be learned?. J Orofac Pain.

[18] Kuzmanovic Pficer J, Dodic S, Lazic V, Trajkovic G, Milic N, Milicic B (2017). Occlusal stabilization splint for patients with temporomandibular disorders: meta-analysis of short and long term effects. PLoS ONE.

[19] Michelotti A, de Wijer A, Steenks M, Farella M (2005). Home-exercise regimes for the management of non-specific temporomandibular disorders. J Oral Rehabil.

[20] Giannakopoulos NN, Katsikogianni EN, Hellmann D, Eberhard L, Leckel M, Schindler HJ (2016). Comparison of three different options for immediate treatment of painful temporomandibular disorders: a randomized, controlled pilot trial. Acta Odontol Scand.

[21] Ottl P, Reiber T, Lange M, Lauer H-C. Klinischer Funktionsstatus der Deutschen Gesellschaft für Funktionsdiagnostik und -therapie (DGFDT) in der DGZMK [Clinical Functional Status of the German Society for Functional Diagnostics and Therapy (DGFDT) in the DGZMK]. Downloaded from the internet: https://www.dgfdt.de/richtlinien_formulare. Accessed 04-06-2021.

[22] Treede RD, Rief W, Barke A, Aziz Q, Bennett MI, Benoliel R (2015). A classification of chronic pain for ICD-11. Pain.

[23] Sabbagh A, Mahony D. Functional diagnostics and AquaSplint Therapy A novel procedure for simple diagnosis and effective therapy of the TMJ/TMD during orthodontic treatment. Australasian Dentist. 2011;9:37–8.

[24] Gedrange T, Sabbagh A, Sabbagh R, Gredes T, Botzenhart U. Patient’s experience and satisfaction to Aquasplint treatment - results of patient’s questioning. EOS Annual Congress 2015.

[25] Srivastava R, Jyoti B, Devi P (2013). Oral splint for temporomandibular joint disorders with revolutionary fluid system. Dent Res J (Isfahan).

[26] Glassman B (2002). The Aqualizer’s role. Dent Today.

[27] Nordstrom D (2000). Craniometer/Aqualizer techniques. Funct Orthod.

[28] Manfredini D, Winocur E, Ahlberg J, Guarda-Nardini L, Lobbezoo F (2010). Psychosocial impairment in temporomandibular disorders patients. RDC/TMD axis II findings from a multicentre study. J Dent.

[29] Bueno CH, Pereira DD, Pattussi MP, Grossi PK, Grossi ML (2018). Gender differences in temporomandibular disorders in adult populational studies: a systematic review and meta-analysis. J Oral Rehabil.

[30] Schmid-Schwap M, Bristela M, Kundi M, Piehslinger E (2013). Sex-specific differences in patients with temporomandibular disorders. J Orofac Pain.

[31] Turner JA, Mancl L, Huggins KH, Sherman JJ, Lentz G, LeResche L (2011). Targeting temporomandibular disorder pain treatment to hormonal fluctuations: a randomized clinical trial. Pain.

[32] Racine M, Tousignant-Laflamme Y, Kloda LA, Dion D, Dupuis G, Choinière M (2012). A systematic literature review of 10 years of research on sex/gender and experimental pain perception - part 1: are there really differences between women and men?. Pain.

[33] Dao TT, LeResche L (2000). Gender differences in pain. J Orofac Pain.

[34] Bagis B, Ayaz EA, Turgut S, Durkan R, Özcan M (2012). Gender difference in prevalence of signs and symptoms of temporomandibular joint disorders: a retrospective study on 243 consecutive patients. Int J Med Sci.

[35] Cooper BC, Kleinberg I (2007). Examination of a large patient population for the presence of symptoms and signs of temporomandibular disorders. Cranio.

[36] Lundh H, Westesson PL, Kopp S, Tillström B (1985). Anterior repositioning splint in the treatment of temporomandibular joints with reciprocal clicking: comparison with a flat occlusal splint and an untreated control group. Oral Surg Oral Med Oral Pathol.

[37] Helkimo M (1974). Studies on function and dysfunction of the masticatory system. II. Index for anamnestic and clinical dysfunction and occlusal state. Sven Tandlak Tidskr.

[38] Conti PC, Corrêa AS, Lauris JR, Stuginski-Barbosa J (2015). Management of painful temporomandibular joint clicking with different intraoral devices and counseling: a controlled study. J Appl Oral Sci.

[39] Speciali JG, Dach F (2015). Temporomandibular dysfunction and headache disorder. Headache.

[40] Gonçalves DA, Bigal ME, Jales LC, Camparis CM, Speciali JG (2010). Headache and symptoms of temporomandibular disorder: an epidemiological study. Headache.

[41] Mottaghi A, Menéndez-Díaz I, Cobo JL, González-Serrano J, Cobo T (2019). Is there a higher prevalence of tinnitus in patients with temporomandibular disorders? A systematic review and meta-analysis. J Oral Rehabil.

[42] Tullberg M, Ernberg M (2006). Long-term effect on tinnitus by treatment of temporomandibular disorders: a two-year follow-up by questionnaire. Acta Odontol Scand.

[43] Wright EF (2007). Otologic symptom improvement through TMD therapy. Quintessence Int.

[44] de Felício CM, Melchior Mde O, Ferreira CL, Da Silva MA (2008). Otologic symptoms of temporomandibular disorder and effect of orofacial myofunctional therapy. Cranio.

[45] Sobhy OA, Koutb AR, Abdel-Baki FA, Ali TM, El Raffa IZ, Khater AH (2004). Evaluation of aural manifestations in temporo-mandibular joint dysfunction. Clin Otolaryngol Allied Sci.

[46] Wichelhaus A, Haas R, Sander F-G, Kreidler J-F (1998). The influence of the spring activator on the mobility of the lower jaw in traumatically injured patients. J Orofac Orthop.

[47] Zhang SH, He KX, Lin CJ, Liu XD, Wu L, Chen J (2020). Efficacy of occlusal splints in the treatment of temporomandibular disorders: a systematic review of randomized controlled trials. Acta Odontol Scand.

[48] Stechman-Neto J, Porporatti AL, Porto de Toledo I, Costa YM, Conti PC, De Luca Canto G (2016). Effect of temporomandibular disorder therapy on otologic signs and symptoms: a systematic review. J Oral Rehabil.

[49] Fricton J (2006). Current evidence providing clarity in management of temporomandibular disorders: summary of a systematic review of randomized clinical trials for intra-oral appliances and occlusal therapies. J Evid Based Dent Pr.

[50] Greene CS (2001). The etiology of temporomandibular disorders: implications for treatment. J Orofac Pain.

[51] Stohler CS, Zarb GA (1999). On the management of temporomandibular disorders: a plea for a low-tech, high-prudence therapeutic approach. J Orofac Pain.

